# Tolerance and dose-volume relationship of intrathoracic stomach irradiation after esophagectomy for patients with thoracic esophageal squamous cell carcinoma

**DOI:** 10.18632/oncotarget.4730

**Published:** 2015-08-05

**Authors:** Qi Liu, Xu-Wei Cai, Xiao-Long Fu, Jun-Chao Chen, Jia-Qing Xiang

**Affiliations:** ^1^ Department of Radiation Oncology, Fudan University Shanghai Cancer Center, Shanghai, China; ^2^ Department of Oncology, Shanghai Medical College, Fudan University, Shanghai, China; ^3^ Department of Radiation Oncology, Shanghai Jiaotong University Chest Hospital, Shanghai, China; ^4^ Department of Thoracic Surgery, Fudan University Shanghai Cancer Center, Shanghai, China

**Keywords:** Clinical Section, esophageal carcinoma, radiotherapy, toxicity, intrathoracic stomach

## Abstract

**Purpose:**

To identify the tolerance of radiation with a high prescribed dose and predictors for the development of intrathoracic stomach toxicity in patients with thoracic esophageal squamous cell carcinoma (SCC) after esophagectomy followed by gastric conduit reconstruction.

**Methods and Materials:**

From 2011 to 2013, 105 patients after esophagectomy were treated with postoperative radiotherapy. The intrathoracic stomach was outlined with the calculation of a dose-volume histogram (DVH) for the initial intended treatment of 6020 cGy or 6300 cGy. The volume of the intrathoracic stomach receiving each dose was recorded at 10-Gy intervals between 10 and 40 Gy and at 5-Gy intervals between 40 and 60 Gy. The grade of toxicities was defined by the National Cancer Institute Common Toxicity Criteria version 4.0.

**Results:**

The mean and maximum doses of the intrathoracic stomach were 2449 ± 986 cGy and 6519 ± 406 cGy, respectively. Sixteen (15.2%) and three (2.9%) experienced Common Toxicity Criteria Grade 2 and Grade 3 acute gastric toxicity. There were no Grade 4 toxicities. Fourteen patients (13.3%) exhibited late gastric complications possibly related to radiation. The volume percent of the intrathoracic stomach receiving at least 50 Gy (V_50_) was strongly associated with the degree of toxicity (*p* = 0.024, respectively). Multivariate analysis of patient and treatment-related factors revealed no other significant predictors of severe toxicities.

**Conclusions:**

The intrathoracic stomach is well tolerated with a high-dose irradiation for patients with esophageal SCC receiving radiotherapy after esophagectomy. A strong dose-volume relationship exists for the development of Grade 2 acute intrathoracic stomach toxicity in our study.

## INTRODUCTION

Existing evidences have indicated that overall survival (OS) could be improved using neoadjuvant chemoradiotherapy followed by surgery for esophageal cancer [1]. However, neoadjuvant therapy might increase the risk of postoperative morbidity or perioperative mortality [2], so a considerable number of patients with local advanced thoracic esophageal squamous cell carcinoma (SCC) in our country perform surgery as their initial treatment. However, sometimes it is difficult to achieve the purpose of complete resection because of surgeons' skills. Moreover, according to previous studies the recurrence rate of SCC is as high as 40%–50% after radical surgery, and locoregional recurrence accounts for more than half of treatment failures [3–4], even among patients with a pathologically complete response to neoadjuvant chemoradiotherapy [5]. Recurrences in supraclavicular and superior mediastinal areas were the most common failures [6–7]. It further necessitates the need for adjuvant therapy to decrease the likehood of local recurrence, especially for patients with positive lymph nodes [8–9]. Radiotherapy(RT) is also a crucial treatment for locoregional failures. Usually, the radiation dose is - as high as possible for patients with limited lesions.

Stomach is the first choice for esophageal replacement following esophagectomy. Recently, gastric tube has gained wide acceptance for esophageal reconstruction, which significantly improve the quality of postoperative life. In our center most of patients received esophagectomy with gastric tube reconstruction and intrathoracic anastomosis via the retrosternal route. As a result, the intrathoracic stomach is often incidentally irradiated in postoperative thoracic radiotherapy. Therefore, when formulating radiation plans for patients who have undergone surgery, the intrathoracic stomach need to be protected to avoid severe complications such as marginal ulcers, bleeding, perforation and anastomotic fistula, as RT-induced injury could occur hours to weeks after the first treatment [10]. Gastroduodenal (GD) tolerance to RT has been investigated in abdominal malignancies [11–12], but the application of these results to esophageal SCC patients requires caution. The reason is there are very few published reports of acute and late RT effects on the intrathoracic stomach, and whether the effects would influence the functions of the gastric substitute is not well known.

Due to technical limitations of 2-dimensional RT, the actual dose distribution of organs at risk could not be analyzed. Currently, developed three-dimensional conformal RT (3D-CRT) has been succeed in providing the possibility of analyzing dose-toxicity relationship and decreasing certain toxicities in esophageal SCC patients. In the present study, various clinical and dose-volume histogram (DVH) parameters were analyzed based on a widely used radiation system in order to identify a potentially safe dose tolerance of the intrathoracic stomach and risk factors for gastric toxicity by observing the radiation-induced adverse side effects in a group of patients from a randomized phase II clinical trial (http://ClinicalTrials.gov website, number NCT01391572) who received thoracic RT after esophagectomy.

## MATERIALS AND METHODS

### Patient population and treatment

**Figure 1:**
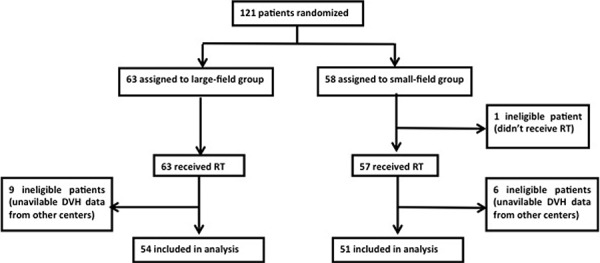
CONSORT diagram for the study RT = radiotherapy.

Between May 2011 and December 2013, patients from 6 centers with esophageal SCC undergoing surgical resection followed by gastric tube reconstruction enrolled our clinical trial. The patients were included if their pathological stage was T_3–4_N_0–3_M_0_ according to the AJCC/UICC TNM staging system (Version 7.0, 2009) and if they did not receive neoadjuvant therapy. Patients without postoperative RT or available DVH data were ineligible (Figure 1).

All patients underwent esophagectomy through right thorax and abdominal incisions and done in esophageal reconstruction. The intrathoracic stomach was formed from the distal aspect of the lesser curvature of the stomach with application of linear staplers. It was created by resection of the lesser curvature and formation of the gastric conduit (4–6 cm in diameter). And then, the tubular stomach was pulled upward to the cervical or aortic arch part through the posterior mediastinal route and performed two-layer anastomosis. All included patients were never diagnosed with gastric ulcers, reflux esophagitis and other serious gastrointestinal diseases that would preclude safe administration of treatment.

Components of the pre-radiation process included the following: a complete history and physical examination; complete blood cell counts; serum biochemical assays; barium esophagram to exclude evidence of gastric perforation, anastomotic fistula, or deep ulceration to the mediastinum; chest CT scans; and ultrasonographic examination to rule out distant metastases in the neck, liver, kidney, spleen, and retroperitoneal lymph nodes.

**Figure 2:**
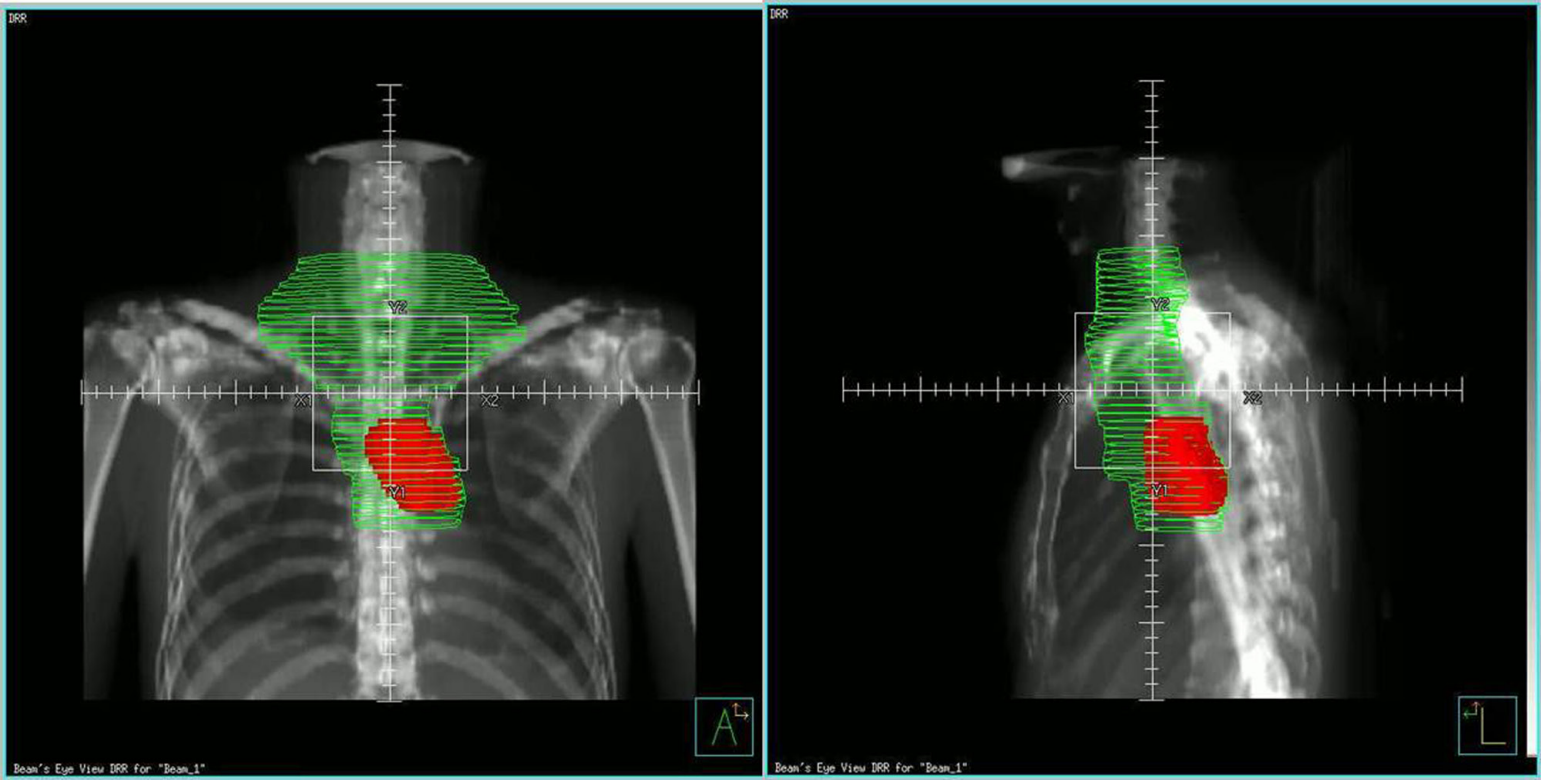
The radiation plan displays on the coronal and sagittal planes Red shading indicates the planning tumor volume (PTV) of small field group (tumor bed area only), the green line outlines the PTV of large-field group including bilateral supraclavicular and upper mediastinal lymphatic drainage area and tumor bed area.

All patients underwent CT-based treatment simulation while supine, and 5-mm-thick images were obtained throughout the entire neck, thorax, and upper abdomen. The clinical tumor volumes (CTVs) encompassed the tumor bed and/or the bilateral supraclavicular and upper mediastinal lymphatic drainage areas. Planning target volumes (PTVs) were defined as the CTV plus a uniform 1-cm margin. According to the protocol for estimating the optimal radiation volume of postoperative radiation, all patients were assigned into either the large-field group (including tumor bed, bilateral supraclavicular and upper mediastinal lymphatic drainage areas) or the small-field group (only the tumor bed area) by random number table. A simultaneous integrated-boost intensity-modulated radiotherapy (SIB-IMRT) technique was used, and treatment plans were generated by the Pinnacle treatment planning system (Philips Medical Systems). Radiation was delivered with 6-MV photons by a linear accelerator. The prescribed doses of the tumor bed area were 60.2 Gy (in 28 fractions of 2.15 Gy/fraction) using a 6-MV X-ray for patients with T3-stage disease or 63 Gy (in 28 fractions of 2.25 Gy/fraction) for patients with T4-stage disease. The lymphatic drainage area in the large-field group was prescribed a dose of 50.4 Gy (Figure 2). The goals were to deliver the prescription dose to at least 95% of the PTV and 95% of the prescribed dose to at least 99% of the PTV. The normal tissue constraints met the following criteria: (1) maximum spinal cord dose ≤ 45 Gy; (2) lung V20 ≤ 25% and mean lung dose (MLD) ≤ 15 Gy; and (3) mean heart dose ≤ 30 Gy. The intrathoracic stomach was not designated as a constrained structure for the original treatment plans. All patients were required to fast during simulation and irradiation.

### Follow-up and diagnosis of gastric toxicity

Follow-up occurred 4–6 weeks after treatment completion and every 3 months thereafter. Complete history and physical examinations, including an evaluation of digestive symptoms, were performed at each visit. Re-examinations included endoscopy, cervical ultrasounds, chest-enhanced CT scans, abdominal ultrasound screening, and, when necessary, bone emission computed tomography (ECT) and positron emission tomography (PET)/CT. Gastric toxicity was defined as the new development of or the aggravation of endoscopic abnormalities, such as erosive gastritis or a gastric ulcer in close proximity to the RT field following RT. Assessment of digestive symptoms was adapted from the Common Toxicity Criteria for Adverse Events, version 4.0.

### Dosimetric evaluation of the intrathoracic stomach

Intrathoracic stomach contour (PTV_sto_) was defined as a three-dimensional expansion of the intrathoracic tubular stomach. DVH were recorded for the lung, heart, spinal cord, intrathoracic stomach and PTV in all patients. The dosimetric parameters from DVHs were as follows: (1) D_max_: maximum dose, (2) D_mean_: mean dose (3) V_dose_: percentage volume receiving more than the irradiated dose and (4) aV_dose_: absolute volume receiving more than the irradiated dose. The range of the intrathoracic stomach volume in 10 Gy (V_10_) to 40 Gy (V_40_), at 10 Gy intervals and in 40Gy (V_40_) to 60Gy (V_60_), at 5 Gy intervals was acquired. Patients with Grade 2 toxicity and above were analyzed further to determine their risk of developing toxicity according to the dose-volume parameters.

### Statistical analysis

The primary endpoint was the occurrence and severity of toxicity. Continuous variables were summarized by descriptive statistics, and categorical variables were tabulated as frequencies and percentages. Dosimetric comparisons between small-field and large-field group were performed with independent sample *t*-tests in all patients. The association between toxicity grade (0–1 vs. ≥ 2) and irradiated stomach volume and other clinical and treatment-associated factors were analyzed by multiple logistic regression. Factors analyzed included the following: age, gender, irradiation volume, anastomotic location, surgery procedure and location of the intrathoracic stomach. The area under the curve (AUC) was calculated to determine the cut-off value of the best predictors. Statistical analyses were performed with Statistical Package for the Social Sciences software (Version 13.0, Chicago, IL, USA). All tests were two-sided, and *P*-values less than 0.05 were considered significant.

## RESULTS

A total of 105 patients with esophageal SCC were identified for analysis. Table 1 lists the patient and tumor characteristics. The median age of patients included in the study was 58 years (range, 40–71). The median follow-up time was 16.0 months (range, 3.0–37.3 months). The cases included 79 (75.2%) cervical anastomoses and 26 (24.8%) aortic anastomoses. The median volume of the intrathoracic stomach was 229.06 ml (73.24–440.50 ml), the D_max_ of PTV_sto_ was 6519 ± 406 cGy, and the D_mean_ was 2449 ± 986 cGy. Among the 51 patients in the small-field group, the D_mean_ and D_max_ of PTV_sto_ were 2062 cGy and 6374cGy, respectively, compared with 2813 cGy and 6657 cGy, respectively among the 54 patients in the large-field group (both *p* < 0.001, respectively). Independent sample *t*-tests found all mean aV_dose_ and V_dose_ parameters but aV_60_ and V_60_ significantly higher in the large-field group than the small-field group (*p* < 0.05, showed in Table 2).

**Table 1:** Patients characteristics

		Large-field	Small-field	Total	*p*
Gender	Male	46	45	91	0.646
	Female	8	6	14	
Length	< = 4cm	33	34	67	0.694
	> 4cm	21	17	38	
Pathological	well	2	3	5	0.823
differentiation	Moderately	33	28	61	
	poorly	18	15	33	
Tumor location	Upper thoracic	3	4	7	0.611
	Middle thoracic	24	18	42	
	Lower thoracic	27	29	56	
T stage	T3	52	44	96	0.067
	T4	2	7	9	
N stage	N0	25	20	45	0.174
	N1–3	29	31	60	
Stomach location	Postmediastinum	25	15	40	0.195
	Left thoracic cavity	4	6	10	
	Right thoracic cavity	25	30	55	
Anastomosis	Cervix	39	40	79	0.461
	Intrathoracic	15	11	26	
Lymphadenectomy	Two-field	49	45	94	0.675
	Three-field	5	6	11	

**Table 2:** Comparison between small-field group and large-field group in dosimetric parameters

	Small-field group	Large-field group	*P*
aV10(ml)	118.48	149.84	0.019
aV20(ml)	92.96	125.01	0.005
aV30(ml)	72.78	104.23	0.001
aV40(ml)	49.62	79.99	0.001
aV45(ml)	41.07	68.14	0.001
aV50(ml)	33.88	56.80	0.001
aV55(ml)	27.15	38.04	0.03
aV60(ml)	22.20	25.03	0.521
V10(%)	50.1	63.3	0.001
V20(%)	39.6	54.2	0.001
V30(%)	31.9	46.6	0.001
V40(%)	22.4	37.7	0.001
V45(%)	18.8	33.2	0.001
V50(%)	15.4	28.6	0.001
V55(%)	12.7	19.8	0.012
V60(%)	10.6	13.5	0.248

Treatment toxicities were classified according to the clinical symptoms. Overall, the maximum acute gastric toxicities encountered during RT were Grade 0 in 82 patients (78.1%), Grade 1 in 4 patients (3.8%), Grade 2 in 16 patients (15.2%) and Grade 3 in 3 patients (2.9%). There were no Grade 4 toxicities and no treatment-related deaths. None of patients required a treatment break because of gastric toxicity. The cases exhibited Grade 2 and above acute digestive reactions, including 2 with abdominal pain (1.9%), 9 with anorexia (8.6%), 8 with sour regurgitation (7.6%), and 4 with gastrectasia (3.8%). All 3 cases with Grade 3 toxicity was anorexia. The median time to the onset of these symptoms was 16 days (range, 1–40 days).

During the follow-up period, 15 (14.3%) patients suffered from grade 2 and above late toxicities, including 7 (6.7%) with severe anastomotic obstruction, 4 (3.8%) with severe gastritis, 2 (1.9%) with remnant gastric ulcer, 1 (1.0%) with gastric bleeding and 1 (1.0%) with bronchial stump gastric fistula. The patients who suffered from gastric bleeding and bronchial stump gastric fistula died from their complications. The median time to the onset of late toxicities was 4.5 months (range, 1.0–12.0 months). Moreover, the incidences of acute (35.2% vs. 29.4%, *p* = 0.527) and late gastric toxicities (18.5% vs. 9.8%, *p* = 0.202) in the large-field group were a little higher than those in the small-field group, but no statistically significant difference was found.

By multiple logistic regression analysis, V_50_ was the only predictive factor for Grade 2 and above gastric toxicity (*p* = 0.024, Table 3). ROC curve analysis showed that the cut-off value of V_50_ was 14.05% (0.815, 95% CI:0.685–0.946; the sensitivity and specificity were 82.4%, and 61.3%, respectively) and that the rates of Grade 2 and above acute and late toxicities were 19.1% for V50 < 14.05% and 34.5% for V50 > 14.05%.

**Table 3:** Multiple analyses of the risk factors related to Grade 2 and above gastric toxicities

Variables		Multiple regression		
		Exp(B)	95% CI	*P* value
Age		1.090	0.999–1.189	0.054
Gender	Male vs. Female	1.774	0.300–10.500	0.527
Irradiation volume	Large field vs. Small field	3.777	0.656–21.751	0.137
Anastomotic location	Cervix vs. Intrathoracic	2.683	0.592–12.147	0.200
Surgery procedure	Two-field vs. Three-field	1.096	0.199–6.044	0.916
Location of intrathoracic stomach	Postmediastinum vs. Thoracic cavity	3.536	0.382–32.76	0.266
T stage	T3 vs.T4	0.541	0.043–6.833	0.635
V10		0.025	0.001–9.890	0.483
V20		2.43	0.000–9.172	0.446
V30		2.615	0.000–5.648	0.704
V40		1.300	0.286–3.331	0.883
V45		6.356	0.358–65.261	0.958
V50		13.815	1.42–134.38	0.024
V55		13.988	0.783–249.78	0.073
V60		7.989	0.304–209.782	0.213
aV10		1.01	0.900–1.134	0.206
aV20		0.913	0.706–1.181	0.252
aV30		1.232	0.901–1.686	0.183
aV40		0.521	0.306–1.127	0.171
aV45		0.573	0.323–1.144	0.224
aV50		0.646	0.358–1.163	0.274
aV55		0.855	0.485–1.506	0.082
aV60		0.984	0.645–1.502	0.060
D_max_		1.000	0.999–1.002	0.688
D_mean_		0.988	0.990–1.006	0.610

D_max_: maximum dose of intrathoracic stomach; D_mean_: mean dose of intrathoracic stomach

## DISCUSSION

Our results show that ≥ grade 2 acute and late gastric toxicity occurred in 18.1% and 14.3% of patients, respectively, and that an RT dose-volume effect for stomach toxicity was shown. V_50_ was the most predictive factor for ≥ grade 2 toxicity for the stomach.

In the era of 3D-CRT, upper digestive tract tolerance to RT has been investigated in abdominal malignancies. The overall incidence of GD toxicity after RT was reported to be between 5.7% and 23.1% in hepatocellular carcinoma [13–15]. The risk of grade 2 and greater GD toxicities was reported to be between 33% and 80% when combined with chemotherapy in pancreatic cancer [11–12, 16]. In two retrospective studies for esophageal cancer, the risk of acute toxicity in the upper aerodigestive tract and stomach was 2.3%–11.9% for the small T portal group and 12%–18.6% for the large T portal group [8, 17], which was similar to our results. Cosset et al. [18] reported severe late gastric complications included ulcers (*n* = 25) and severe gastritis (*n* = 2) among 516 patients with Hodgkin's disease treated by RT close to 40 Gy. Chen et al. also reported the risk of late complications in their study, 1 of 355 patients experienced grade 2–3 gastric bleeding and another 4 patients experienced grade 5 gastric bleeding after postoperative radiation. In a phase II trial of postoperative concurrent chemoradiotherapy, 8% patients experienced grade 3–4 upper digestive tract toxicity, and 6% patients required an unplanned hospitalization [19]. The use of concurrent chemotherapy, differences in RT volume and prescribed dose, and selection bias for the study population may explain the different rates of modest complications in these studies. The irradiated dose in our study was higher when being converted into the biologically effective dose (BED), while partial patients' irradiated volumes were smaller than in previous studies.

In early reports, the gastric ulceration and perforation rates were 4% and 2% vs. 16% and 14% after doses <50 Gy vs. ≥ 50 Gy [10]. Emami et al. [20] demonstrated the tolerance dose for late gastric ulceration was 50, 55, and 60 Gy for the whole stomach, 2/3 of the stomach, and 1/3 of the stomach, as a suggestion for TD5/5 (the probability of 5% complication within 5 years). However, they did not offer estimates to predict acute toxicities. Currently, for stomach tolerance, the current Quantitative Analysis of Normal Tissue Effects in the Clinic (QUANTEC) report recommends dose constraints of the stomach for patients with abdominal tumors as follows: whole stomach 50 Gy (range of maximum 45–54 Gy) and partial volume dose limits of 2% V_50_–10% V_45_. [21]. However, these data may not be applicable for esophageal SCC patients because (1) anatomical structures change after surgery, generally causing some scar tissue in the thoracic and peritoneal cavity; (2) intrathoracic stomach tube for upper digestive tract reconstruction contributes to the development of digestive diseases; and (3) the prescribed dose of thoracic radiation always exceeds 50 Gy, even more than 60 Gy. A dose of 50 Gy has endured as a broad dose limit guideline when irradiated fields encompass a large portion of the stomach, albeit with rather limited support from actual published data [10]. However, nearly all of the patients in our study received intrathoracic stomach irradiation with a D_max_ more than 60 Gy. The mean V_60_ for the group was 12.1%, which was much higher than the QUANTEC standards. According to the study of Emami et al., 60 Gy of 1/3 stomach was tolerant for TD5/5; therefore, we believed the risk of severe toxicities due to RT in our study would not be higher than the existing data. Meanwhile, we think it is still important to ensure that hotspots are minimized when using most conformal techniques.

Some of the previously summarized studies reported that RT toxicity was also related to irradiated volume and preformed dose-volume analysis for the stomach. Nakamura et al. [12] reported that V_50_ of the stomach ≥ 16 cm^3^ may be the best predictor for ≥ grade 2 acute gastrointestinal toxicity. Kim et al. showed a dose-volume analysis of GD toxicity in cirrhotic patients with HCC and suggested that V_35_ ≥ 5% could predict ≥ grade 3 GD toxicity [22]. However, there is a lack of data on the evaluation of toxicity based on dose-volume analysis for the intrathoracic stomach using DVH parameters. In the current study, we confirmed the dose-volume effect for digestive toxicity. We found that V_50_ for the intrathoracic stomach was the most predictive factor for ≥ grade 2 gastric toxicity. Patients in the large-field group received a greater range of radiation exposure with the same prescribed dose, meaning that the volume of the intrathoracic stomach irradiated was higher than that in the small-field group. Thus, the large-field group had a higher risk of radiation toxicity than the small-field group, though statistical significance was not reached. In fact, literature on RT-induced stomach toxicity is relatively sparse, with insufficient data to arrive at firm dose-volume constraints for partial volume irradiation.

There were several limitations in our study. First, the volume of the stomach is variable; therefore, errors in our data were inevitable. However, patients were requested to avoid large meals or carbonated beverages before simulation and treatment to minimize variability in the volume and location of the stomach. Second, we record the toxicities mainly based on patients' symptoms rather than objective examinations. Third, selection bias may have influenced the results. If more patients were enrolled and observed for a longer time period, the values might change. Thus, a larger study is necessary to verify our results.

Therefore, it is acceptable to keep the maximum point dose to the intrathoracic stomach at more than 60 Gy or less for acute and late gastric toxicity for patients with esophageal cancer treated with radiotherapy after esophagectomy. A strong dose-volume relationship exists for the development of Grade 2 acute intrathoracic stomach toxicity in our study. Further studies are necessary to clarify the dose-volume relationship for intrathoracic stomach toxicity and to determine its dose constraint.
